# Accuracy of clinical criteria and an immunochromatographic strip test for dengue diagnosis in a DENV-4 epidemic

**DOI:** 10.1186/s12879-016-1368-7

**Published:** 2016-01-29

**Authors:** Sibelle Nogueira Buonora, Sonia Regina Lambert Passos, Cleber Nascimento do Carmo, Fernanda Moisés Quintela, Diana Neves Rodrigues de Oliveira, Flavia Barreto dos Santos, Yara Hahr Marques Hökerberg, Rita Maria Ribeiro Nogueira, Regina Paiva Daumas

**Affiliations:** 1 Laboratory of Clinical Epidemiology, Evandro Chagas National Institute of Infectious Diseases, Oswaldo Cruz Foundation, Rio de Janeiro, Brazil; 2 National School of Public Health, Oswaldo Cruz Foundation, Rio de Janeiro, Brazil; 3 Germano Sinval Faria Teaching Primary Care Center, National School of Public Health, Oswaldo Cruz Foundation, Rio de Janeiro, Brazil; 4 Flavivirus Laboratory, Oswaldo Cruz Institute, Oswaldo Cruz Foundation, Rio de Janeiro, Brazil

**Keywords:** Dengue, Accuracy, Sensitivity, Specificity, Diagnosis, Point-of-care systems, NS1

## Abstract

**Background:**

Early diagnosis of dengue infection is important for decision-making and timely implementation of therapeutic measures. Although rapid NS1 assays have been used for dengue diagnosis since 2008, their performance in DENV-4 cases has not yet been fully assessed.

**Methods:**

We evaluated the accuracy of NS1 Bioeasy™ immunochromatographic strip test and of three clinical criteria for dengue diagnosis. Patients presenting at an emergency care center within 72 h of an acute febrile illness during the 2013 DENV-4 epidemic in Rio de Janeiro were consecutively enrolled for clinical and laboratory evaluation. We classified patients as suspected dengue or not according to three clinical criteria: WHO 2009, WHO 1997, and INI-FIOCRUZ. Dengue diagnosis was defined by RNA detection using RT-PCR and the negative cases were negative for all dengue serotypes and also Platelia™ NS1 ELISA. We obtained accuracy indices for NS1 Bioeasy™ alone and in combination with the clinical criteria.

**Results:**

RT-PCR for DENV-4 was positive in 148 out of 325 patients. Positive likelihood ratio, sensitivity, and specificity of NS1 Bioeasy™ with WHO 2009, WHO 1997, and INI-FIOCRUZ criteria were 22.6 (95 % CI 7.2–70.6), 40.6 % (95 % CI 32.3–49.3), and 98.2 % (95 % CI 94.9–99.6); 18.3 (95 % CI 6.8–49.2), 44.2 (95 % CI 35.8–52.9), 97.6 (95 % CI 94.0–99.3); 26.2 (95 % CI 6.5–106.5), 29.7 (95 % CI 22.4–37.8), 98.9 (95 % CI 96.0–99.9), respectively. WHO 1997 clinical criteria presented high sensitivity to rule out disease, but extremely low specificity. INI-FIOCRUZ had moderate sensitivity and specificity, and could target a group to a more specific test.

**Conclusions:**

Although the large rates of false negative results using NS1 Bioeasy™ rapid test advise against its use for triaging (rule out) purposes in DENV-4 epidemics, it could be used as a confirmatory tool in a bedside algorithm.

## Background

Dengue is an acute viral febrile disease mainly transmitted by the *Aedes aegypti* mosquito. Dengue virus has four serotypes (DENV 1-4) defined by phylogenetic and antigenic characteristics. The immunity resulting from infection is serotype-specific and does not protect the individual against other serotypes [1].

A recent study estimated that about 390 million cases of dengue occurred in 2010, which is more than three times the World Health Organization (WHO) estimates for that same period [2]. However, only 96 million were symptomatic. Brazil has the highest dengue reporting rates worldwide and between 2000 and 2007, over three million cases were reported in the country, corresponding to approximately 60 % of cases in the Americas [3].

In Brazil, dengue outbreaks have occurred yearly since 1986 and during epidemic seasons the entire health care system is overwhelmed by a three to four-fold increase in patient visits. In the state of Rio de Janeiro, epidemics occurred in 1986 [4], 1990 [5], 2000 [6], and 2008 [7] by DENV-1, DENV-2, DENV-3, and by the reemergence of DENV-2, respectively, usually followed by dissemination to other regions of the country. DENV-4 was first identified in this Brazilian state in 2011 [8] and accounted for 218,000 reported cases in the 2013 epidemic.

Early diagnosis is critical because some patients may progress from a mild to a severe disease in a short period of time [9, 10]. Repeated monitoring of platelet count and hematocrit is recommended, as an abrupt decrease in platelet count is a warning sign and a significant hematocrit increase is an indirect sign of plasma leakage [7]. In 1997, WHO proposed as a dengue clinical case a patient that presented with fever and two or more of the following: headache, retro-orbital pain, myalgia, arthralgia, rash, hemorrhagic manifestations, and leukopenia [11]. However, due to the low specificity (36 %) of these criteria [12], a new set was proposed in 2009 that grouped myalgia and arthralgia in body aches, included nausea and vomiting, and added some warning signs and symptoms [1]. The 80 % sensitivity and 57 % specificity of the newly proposed WHO criteria [12], supports the importance of laboratory confirmation.

A previous study carried out at the Evandro Chagas National Institute of Infectious Diseases, Oswaldo Cruz Foundation (INI-FIOCRUZ) in Rio de Janeiro analyzed clinical and hematological data in ambulatory febrile patients and derived another diagnostic set of clinical criteria for dengue diagnosis [13]. In this study population the prediction rule, based on the presence of conjunctival hyperemia and leukocyte count, shown 81 % sensitivity and 71 % specificity [13].

Dengue laboratory diagnosis can be performed directly, by identifying the virus or its components, or indirectly, through serological tests detecting antibodies produced against the virus. The sensitivity of each method relies on disease duration at the moment of the clinical specimen collection [14]. The indirect methods are the more commonly used but have limited usefulness in the acute dengue diagnosis. IgM peaks occur around the third to fourth day of disease onset, and therefore a second clinical specimen collection is needed around day 14 to confirm the IgM rise and conclusively diagnose the disease [15]. However, in scenarios where the prevalence of secondary dengue is high, such as in Rio de Janeiro State, the duration and magnitude of the IgM response is reduced possibly impairing the accuracy of this serologic parameter [16]. Furthermore, pairing IgM are usually not point-of-care techniques, limiting their usefulness in epidemic scenarios.

During the febrile phase, detection of viral RNA or nonstructural protein-1 (NS1) are the main methods for the disease diagnosis. However, detection of viral RNA by reverse transcriptase-polymerase chain reaction (RT-PCR) is relatively complex and expensive, and is therefore not feasible in a number of health care settings, particularly in epidemic situations. For this reason, the identification of NS1 has been suggested as an alternative because it is present in the virus membrane, is highly stable and is secreted in the human serum during the early phase of dengue infection [15]. The sensitivity of the NS1 ELISA is reported to be greater than 90 % in primary infections and varies from 60 to 80 % in secondary infections [17, 18].

The rapid detection of dengue NS1 by immunochromatographic methods represents a potential breakthrough for laboratory case confirmation at early stages of disease in settings with limited infrastructure because they require minimal laboratory expertise and provide results within 15 to 30 min of specimen reception [4, 15, 19]. This laboratory technique, first described in 2000 and widely used since 2008, has now been tested in all dengue scenarios, especially in DENV-1 and DENV-3 scenarios [12, 15]. However, DENV-4 samples are still underrepresented [15, 18, 20, 21].

Ferraz, et al. [22], when evaluating mainly Brazilian DENV-1 samples by three different immunochromatographic assays, identified the NS1 Bioeasy™ as the one with higher sensitivity (63 %) despite the high specificity presented by all three (100 %). Another Brazilian study evaluating accuracy parameters of 4 immunochromatographic tests in a panel of acute DENV-1 to DENV-3 concluded that NS1 Bioeasy™ presented an overall 68 % specificity and a 90 % sensitivity which could reach 95 % in a DENV-1 setting [23].

A simple and accurate test to diagnose acute dengue cases in outpatient healthcare settings is still required. Considering this issue, we evaluated the diagnostic accuracy of three clinical dengue diagnostic criteria (WHO 1997, WHO 2009, INI-FIOCRUZ) and NS1 Bioeasy™ immunochromatographic test in an urgent care center during a DENV-4 epidemic in the city of Rio de Janeiro, Brazil.

## Methods

### Ethical statement

This prospective cross-sectional diagnostic accuracy study is reported according to the Standards for Reporting of Diagnostic Accuracy Study (STARD) Guideline [24] and was approved by the Research Ethics Committee of the Evandro Chagas National Institute of Infectious Diseases-FIOCRUZ, CAAE 0066.0.009.000-11, on March 23, 2012.

### Enrollment, data, and specimen collection

We conducted the study in the city of Rio de Janeiro, Rio de Janeiro, Brazil, from March to April 2013, during an ongoing dengue epidemic. Adult patients (age >18 years) attending a public urgent care center (Unidade de Pronto Atendimento-UPA 24H) within 72 h of onset of an acute febrile illness without an evident focus of infection were eligible for the study.

A trained nurse obtained informed consent and prospectively enrolled the patients during business hours 5 days a week. An infectious disease physician collected data on demographic characteristics, symptoms and physical signs using a summarized version of a previously published and tested semi-structured questionnaire [25]. The tourniquet test was not performed and only spontaneous hemorrhagic manifestations were recorded.

We evaluated all patients according to WHO 1997, WHO 2009, and INI-FIOCRUZ clinical criteria defined elsewhere [1, 11, 13, 26].

Blood samples were collected for complete blood count and specific dengue tests including NS1 Bioeasy™, RT-PCR, Panbio® dengue IgM capture ELISA and dengue IgG ELISA, and Platelia™ Dengue NS1 Ag-ELISA. Acute dengue-4 cases were confirmed by RT-PCR. Serum samples were sent to the Flavivirus Laboratory, a regional reference laboratory for dengue and yellow fever at the Oswaldo Cruz Foundation (FIOCRUZ) where they were frozen at −70 °C until using for dengue specific laboratorial tests.

### Index tests

#### NS1 strip test

All febrile cases were tested by the NS1 Bioeasy™ immunochromatographic strip test (Bioeasy™, Standard Diagnostics INC, Korea). This rapid test was chosen because it has already been used in the state of Rio de Janeiro and in other Brazilian states during dengue outbreaks. However, its performance had not been previously assessed. According to the manufacturer’s instruction, the test can be stored at room temperature and can adequately detect the NS1 within 15 min using three drops of whole blood, plasma or serum.

In the immunochromatographic strip test, the NS1 antigen present in the sample will complex with the gold colloidal particles coated with anti-NS1 antibodies. After migration, the complexes will be captured by anti-NS1 antibodies at the test line where a line will appear. The presence of two lines in the cassette’s window means a positive result, whereas the absence of the second line means a negative result with an adequate control line, which ensures the test is working properly.

#### Clinical criteria

Patients were classified as suspected dengue cases according to three sets of clinical criteria described as follows:

*WHO 1997*: fever with two or more of the following: headache, retro-orbital pain, myalgia, arthralgia, rash, hemorrhagic manifestations, and leukopenia [11]; *WHO 2009*: fever with two or more of the following: nausea/vomiting, rash, pain, leukopenia, and any of the following warning signs: abdominal pain, persistent vomiting, edema, mucosal bleeding, lethargy, hepatomegaly or hemoconcentration associated with a sudden drop in platelet count [1]; and *INI*-*FIOCRUZ*: presence of conjunctival redness with leukocytes less than 7500/mm^3^ or less than 3760 leukocytes/mm^3^ independently of other signs or symptoms [13].

Hemoconcentration in a single sample was defined by a hematocrit >53 % in males or 48 % in females [27]. Leukopenia was defined as a total leucocyte count ≤4500 cells/mm^3^ [27].

### Reference tests

#### Dengue viral RNA

In order to identify dengue viral RNA and specifically dengue 4 serotype, we performed RT-PCR according to the protocol described by Lanciotti et al. [28]. This highly specific protocol suggested by WHO [1] was considered the “gold standard” method for dengue-4 case confirmation in this study. Briefly, consensus primers (D1-5′-TCAATATGCTGAAACGCGGAGAAACCG-3′) and D2 (5′-TTGCACCAACAGTCAATGTCTTCAGGTTC-3′) were annealed to any of the four dengue serotypes to amplify a 511-bp product in a reverse transcriptase-polymerase reaction. After a second round of amplification (nested PCR) with type-specific primers (TS1 [5′- CGTCTCAGTGATCCGGGGG- 3′], TS2 [5′-CGCCACAAGGGCCATGAACAG-3′], TS3 [5′-TAACATCATCATGAGACAGAGC-3′] and TS4 [5′-CTCTGTTGTCTTAAACAAGAGA-3′]), DNA products specific for each dengue virus serotype were generated.

#### NS1 antigen detection by ELISA

The Platelia™ Dengue NS1 Ag-ELISA (BioRad Laboratories, France) was used for NS1 antigen capture according to the instructions by the manufacturer.

#### Immunologic markers

We performed the Panbio® dengue IgM Capture ELISA (Alere™, Minas Gerais, Brasil) and Dengue Virus IgG DxSelect™ ELISA (Focus Diagnostics, California, USA) according to the manufacturers’ instructions for the qualitative detection of anti-DENV IgM and anti-DENV IgG antibodies, respectively.

### Case definition

Since we aimed to describe only dengue 4 cases, a dengue (D4) case was defined as a patient confirmed with DENV-4 by RT-PCR. Furthermore, acute DENV-4 cases were classified as primary or secondary infections according to the absence or presence of anti-dengue IgG.

A non dengue (ND) case was defined as a patient with negative results for all dengue biomarkers (viral genome from any serotype, anti-dengue IgM and NS1).

Patients with negative RT-PCR and Platelia™ Dengue NS1 Ag-ELISA and/or dengue IgM positive results were considered indeterminate [29]. Indeterminate cases and patients with dengue infection by serotypes other than DENV-4 were excluded.

Laboratory personnel performing one test were blinded to the results of other tests.

### Data processing and analysis

All clinical and laboratory data were collected and recorded in a database using the EpiData^©^ 3.1 software [30]. Exploratory analysis was performed using the SPSS^©^ v 17.0 software (SPSS Inc., Chicago, Illinois). MedCalc^©^ 14.8.1 program was used to calculate 95 % confidence intervals (CI) for sensitivity, specificity, positive predictive value (PPV), negative predictive value (NPV), and likelihood ratios of NS1 Bioeasy™ and dengue clinical criteria.

A sample size of 137 positive dengue patients was determined as necessary to estimate, with 95 % confidence level, a sensitivity of 85 % with an absolute error of 6 %. Considering a prevalence of 40 % among febrile patients, 342 febrile subjects should be evaluated.

### Role of funding source

The Brazilian National Council of Scientific and Technological Development (CNPq) funded this study (PROEP 402068/2012, REBRATS 401366/2013-8 and Grant level 2 CNPq 311414/2013-3); however, the funder was not involved in the design, sample handling, analysis, data interpretation, conclusions or decision to publish.

## Results

**Fig. 1:**
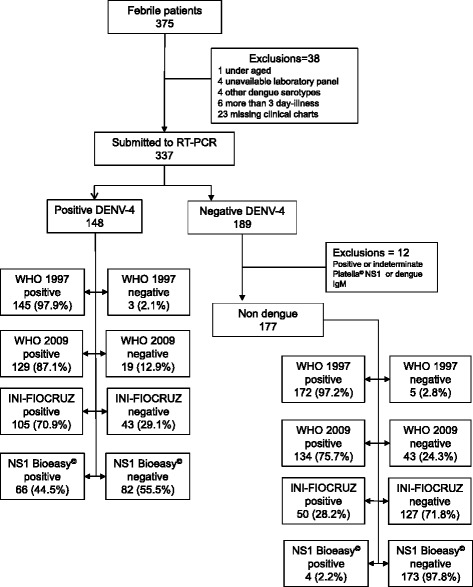
STARD flow diagram of febrile Rio de Janeiro, March–April, 2013

In this study, we evaluated a total of 375 acutely febrile patients. Of those, 38 (10.1 %) were excluded, for reasons shown in Fig. 1. For this reason, 12 patients in the RT-PCR negative group were excluded for presenting positive or indeterminate results for specific dengue IgM or Platelia™ Dengue NS1 Ag-ELISA (Fig. 1).

From the 325 patients included in the study, 148 (45.5 %) had confirmed DENV-4 infection. No significant differences in age and gender distributions were observed between D4 and ND groups. The hematocrits were similar in both groups, but the platelet and leukocyte counts were lower among D4 patients (Table 1). The median time from onset of symptoms and medical care was 2 days in both groups.

**Table 1:** Epidemiological characteristics of 325 febrile patients, Rio de Janeiro, March–April, 2013

	Median (IQR)		
	Dengue 4 (*N* = 148)	Non dengue (*N* = 177)	*p*-value
Age in years	36.6 (23.1–50.1)	36.5 (23.0–50.0)	0.99
Hematocrit (%)	42.6 (40.3–45.0)	42.2 (40.0–44.0)	0.45
Leucocyte counts (cells/mm^3^)	4251 (3400–4667)	7518 (5467–7900)	<0.05
Platelet counts (cells/ mm^3^)	217.812 (187,667–236,667)	256,4945 (221,000–284,667)	<0.05

*IQR* interquartile range

The secondary infections, defined by anti-dengue IgG detection in the D4 group, were characterized in 124/148 (83.7 %) patients, and the NS1 strip test sensitivity was lower (42 %) in secondary (52/124) than in primary dengue cases (62.5 %; 15/24), *p* = 0.05.

We also tested the agreement between the NS1 Bioeasy™ test and the Platelia™ Dengue NS1 Ag-ELISA. The overall agreement, positive and negative concordances between the tests were 84.1, 83.5 and 84.7 %, respectively.

Clinical and laboratory features of eligible patients are shown in Table 2. No sign or symptom alone was able to predict dengue, although leukopenia and thrombocytopenia were more frequent in D4 cases. Leukocyte counts below 4500/mm^3^ were almost eight times more likely to be present in D4 cases. In our study population, we did not find warning signs such as nuchal rigidity, altered consciousness, and ascites.

**Table 2:** Clinical and laboratory features of 325 febrile patients, Rio de Janeiro, March–April, 2013

	Dengue 4 (*N* = 148)		Non dengue (*N* = 177)		OR (95 % CI)
	*N*	%	*N*	%	
Clinical					
Exanthema	28	18.9	21	11.8	1.7 (0.9–3.2)
Conjunctival hyperemia	75	50.6	72	40.6	1.5 (1–2.3)
Lethargy^a^	142	95.9	168	94.9	1.3 (0.4–3.6)
Joint pain	110	74.3	128	72.3	1.1 (0.7–1.8)
Pain	143	96.6	171	96.6	1.0 (0.3–3.4)
Retro-orbital pain	105	70.9	124	70.0	1.0 (0.6–1.7)
Nausea/vomiting	99	66.9	120	67.7	1.0 (0.6–1.5)
Edema^a^	3	2.0	4	2.2	0.9 (0.2–4.0)
Liver enlargement^a^	4	2.7	6	3.3	0.8 (0.2–2.9)
Persistent vomiting^a^	35	23.6	48	27.1	0.8 (0.5–1.4)
Myalgia	134	90.5	161	90.9	0.9 (0.4–1.9)
Abdominal tenderness^a^	47	31.7	68	38.4	0.7 (0.5–1.2)
Bleeding^a^	12	8.1	25	14.1	0.5 (0.3–1.1)
Headache	133	89.8	169	95.4	0.4 (0.2–1. 0)
Laboratory					
Leukopenia	95	64.1	33	18.6	7.8 (4.7–13.0)^b^
Thrombocytopenia	17	11.4	8	4.5	2.7 (1.1–6.5)^b^
Anti-dengue IgG	123	83.1	161	90.9	0.5 (0.3–1.0)

*OR* odds ratio, *CI* confidence interval

^a^warning signs; Leukopenia = leucocyte count <4,500cells/mm^3^; thrombocytopenia = platelet count <150,000 cells/mm^3^

^b^significant odds ratio

Using the WHO 1997 diagnostic criteria, a total of 145 (97.8 %) D4 and 172 (97.2 %) ND were clinically classified as dengue suspected cases, with a sensitivity of 97.8 % and specificity of 2.8 %. The WHO 2009 criteria clinically classified as dengue 129 (87.1 %) D4 and 134 (75.7 %) ND with 87.1 % sensitivity and 24.3 % specificity. The INI-FIOCRUZ clinical prediction rule classified as dengue suspected 105 (70.9 %) D4 patients but also 50 (28.2 %) ND, with a sensitivity of 70.9 % and specificity of 71.8 % (Table 3).

**Table 3:** Accuracy parameters for DENV-4 in 325 febrile patients, Rio de Janeiro, March–April, 2013

	Sensitivity % (95 % CI)	Specificity % (95 % CI)	LR+ (95 % CI)	LR− (95 % CI)	PPV (95 % CI)	NPV (95 % CI)	OR (95 % CI)
WHO 1997	97.8 (93.8–99.6)	2.8 (1.0–6.9)	1.0 (1.0–1.1)	0.7 (0.2–3.0)	45.6 (39.9–51.5)	62.5 (24.5–91.5)	1.4 (0.3–6.0)
WHO 2009	87.1 (80.3–92.1)	24.3 (18.1–31.6)	1.1 (1.0–1.3)	0.5 (0.3–0.9)	48.8 (42.4–55.2)	69.5 (56.1–80.8)	2.2 (1.2–4.0)
INI-FIOCRUZ	70.9 (63.2–78.6)	71.8 (64.5–78.4)	2.5 (2.0–3.3)	0.4 (0.3–0.5)	67.6 (59.5–74.9)	75.3 (68.0–81.7)	6.4 (3.9–10.4)
NS1	44.5 (36.4–53.3)	97.8 (94.2–99.4)	19.5 (7.3–52.2)	0.6 (0.5–0.7)	94.1 (85.6–98.4)	68.3 (62.1–74.0)	34.4 (12.1–97.9)
WHO 1997 and NS1	44.2 (35.8–52.9)	97.6 (94.0–99.3)	18.3 (6.8–49.2)	0.6 (0.5–0.7)	93.9 (85.0–98.3)	67.8 (61.4–73.7)	32.1 (11.3–91.4)
WHO 2009 and NS1	40.6 (32.3–49.3)	98.2 (94.9–99.6)	22.6 (7.2–70.6)	0.6 (0.5–0.7)	94.9 (85.9–98.9)	66.7 (60.4–72.5)	37.3 (11.3–122.9)
INI-FIOCRUZ and NS1	29.7 (22.4–37.8)	98.9 (96.0–99.9)	26.2 (6.5–106.5)	0.7 (0.6–0.8)	95.6 (84.9–99.5)	63.2 (57.2–68.9)	36.9 (8.7–155.5)

*LR*+ positive likelihood ratio, *LR*− negative likelihood ratio, *PPV* positive predictive value, *NPV* negative predictive value, *OR* odds ratio, *CI* confidence interval

The NS1 Bioeasy™ immunochromatographic strip test alone was more accurate than the clinical criteria (OR = 34.4) and showed high specificity (97.8 %). However, the test sensitivity was lower (44.5 %). The test incorrectly classified as ND more than half of dengue patients (55.2 %). With these results almost a third of the patients with a negative NS1 Bioeasy™ test in our setting were in fact confirmed dengue cases (PPV = 68.3 %).

Combining the NS1 test with any clinical criteria did not improve the sensitivity or alter the high false negative rates, which remained similar to those obtained by the test alone. But all the evaluated clinical criteria could be complemented by this test resulting in an almost perfect specificity in a clinical setting.

## Discussion

This is the first study we are aware of that assesses the combined accuracy of dengue clinical criteria and a NS1 point-of-care immunoassay for early diagnosis in outpatients during a DENV-4 epidemic. The WHO 1997 criteria was better to rule out the disease. When the INI-FIOCRUZ clinical criteria was positive, then the NS1 rapid test should be done. Patients with a positive strip test should be treated as dengue cases; however, negative results should be monitored for dengue or other acute febrile illnesses. As the clinical diagnosis lacks specificity, a definitive dengue case may need laboratory confirmation.

According to Lima, et al. [17], RT-PCR used in the present research showed 90 % sensitivity and more than 95 % specificity on the second day after the onset of disease, the same median time obtained in our sample. It is reasonable to consider this as a good diagnostic tool to confirm dengue fever. However, considering false negative results, we excluded 12 patients with negative RT-PCR and a positive or indeterminate IgM or Platelia™ NS1 results in which we could not identify the serotype.

At the time the study population was evaluated, there were no reports of any other Flavivirus circulating simultaneously in the city of Rio de Janeiro that could interfere in dengue laboratory results. We believe that we obtained a true non-dengue set of patients.

We also evaluated the ability of hematological parameters and individual signs and symptoms to discriminate dengue from non-dengue. We found that leukopenia was more frequent in D4 than in ND, although the overlapping range between the two groups prevented an adequate discrimination between them. The relevance of leukopenia as a discriminant feature of dengue infection has been previously documented, although the cutoff values may vary [13, 31–34].

The platelet counts were lower in the D4 group, although true thrombocytopenia was infrequent in both groups. Similar to findings in other studies [22, 35], there was no significant difference in hematocrit between groups. This may be because both hemoconcentration and platelet drop are usually not seen in the first days of disease [34]. Repeated monitoring of the platelet count and hematocrit is recommended, as an abrupt fall in platelet count is proposed as a warning sign, and a significant hematocrit increase is an indirect sign of plasma leakage [10, 34].

Gan, et al. [36] studied 256 adults in Singapore and reported 87.1 % sensitivity and 26 % specificity for the WHO 2009 criteria, similar to the present research. However, the 20 % sensitivity using the WHO 1997 criteria was far different from those obtained in our study.

Due to its lower sensitivity (70.9 %), the INI-FIOCRUZ clinical criteria does not seem very promising as an exclusive screening alternative. However, it was much more specific than both WHO criteria and showed the best clinical performance for classifying suspected dengue cases. It could be proposed (and tested in a different population) as a screening tool to further confirm using more expensive and time consuming specific laboratory tests.

Among the signs and symptoms in the three sets of clinical criteria, none was significantly more frequent among D4 patients. However, conjunctival hyperemia, a sign used in the INI-FIOCRUZ criteria, had a performance similar to classic signs, such as exanthema. Although not sufficiently accurate to be used as a discriminant feature alone, this was an important finding because this clinical sign is not included in the WHO criteria. At least three other studies have reported similar results, both in febrile outpatients in early disease stages [13, 35, 37].

As described by Leo, et al. [38] and Paranavitane, et al. [39] our study targeted early dengue disease in outpatient setting, therefore we could not assess warning signs such as nuchal rigidity, altered consciousness, and ascites, abrupt platelet count fall or hemoconcentration, that occur later during the disease.

The WHO 2009 dengue criteria improved rates of correct identification of severe dengue cases, aiding therapeutics and avoiding unnecessary deaths [30]. However, it did not prove to be much more accurate (sensitive) for screening or clinically diagnosing dengue 4 than the WHO 1997 version. INI-FIOCRUZ criteria showed the best-balanced performance and could enhance specificity being used previously to the NS1 rapid test in selecting a group of patients for this more specific test.

The NS1 Bioeasy™ showed excellent specificity (97.8 %) in the study population, which agrees with previous reported results in different countries [15, 40]. However, we found a substantially lower sensitivity (44.5 %) of Bioeasy™ NS1 immunoassay in our DENV-4 patients compared to the 61.3 and 90 % sensitivity described respectively by Ferraz, et al. [22], and Silva, et al. [23] in DENV-1 and DENV-3 scenarios. Lower sensitivity to DENV-4 compared to the other serotypes has also been documented by Pal, et al. [15], varying from 42 to 58 % depending on the test used.

Differing performances according to serotype were also recently described in a meta-analysis evaluating two NS1 ELISA tests for early dengue detection [18]. The study concluded that Platelia™ NS1 and NS1 Panbio® tests showed lower pooled sensitivity for DENV-4 (58 and 37 %, respectively) [18]. The interpretation given by some authors includes the NS1 gene polymorphism associated with immunological epitopes, a low NS1 concentration in DENV-4 cases [20, 41], or low overall viremia [21]. Nevertheless, this latter hypothesis was not confirmed by Allonso, et al. [20] in a recent Brazilian study.

As reported in other studies on NS1 detection using conventional ELISA or rapid tests, we observed lower NS1 Bioeasy™ sensitivity in secondary (42 %) compared to primary (62.5 %) dengue cases [9, 12, 15, 20]. As most of the cases (83.7 %) were secondary infections, this might have contributed to the poor performance of the test in our population. This low performance probably occurred due to a rapid clearance in secondary cases [15] and/or as a result of antigen-antibody complexes that disrupt the test targets impeding the ability of the test to detect free NS1 antigen [42, 43]. Efforts to dissociate immune complexes by acid or heat treatment can probably enhance the assays sensitivities [44].

The high PPV (94.1 %) obtained in this 45.5 % dengue prevalence scenario, similar to that obtained by Pal, et al. [15], indicates that individuals testing positive on NS1 do not require another confirmatory test. The performance on specificity qualifies this test, as a confirmatory one, to streamline the decision to treat the correctly identified dengue patients in the clinical setting. It should be used just after the onset of symptoms in order to avoid complications and deaths and to organize the flow of care at the facility. This rapid test might also be useful for virologic surveillance purposes to select serum samples for RNA detection by PCR.

However, although highly specific, the NS1 rapid test yields high false negative rates (55.5 %) among confirmed DENV-4 patients, and its low sensitivity argues against its incorporation as a screening diagnostic tool in clinical practice. Recently, the Rio de Janeiro State Health Department decided not to include the test for dengue screening in outpatient settings. Its use has been restricted to evaluate critically ill hospitalized patients.

Our study has some limitations: a small sample of primary dengue patients and the absence of the tourniquet test, a WHO sign criteria, considered a painful and lengthy procedure with poor patient compliance. We were also not able to reevaluate the patients in the convalescent period due to the pragmatic diagnostic study outline and the dynamics of the Rio de Janeiro city’s health care system. Although the gold standard for dengue diagnosis would be pairing specific dengue IgM in acute and convalescent period, as proposed by WHO [11], recent studies have also exclusively used PCR as the reference test to evaluate early phase dengue [45], to evaluate clinical algorithms [46] or ELISA and NS1 tests [15].

## Conclusions

The development of more sensitive point-of-care tests for dengue diagnosis to act as a screening tool remains a challenge, especially for endemic countries, where the prevalence of secondary cases is high. In summary, we proposed a clinical-laboratory algorithm which combines the INI-FIOCRUZ and the WHO 1997 clinical criteria, in order to select ambulatory suspected early dengue cases (within 3 days of onset of disease) eligible for NS1 strip testing. The patients that test positive should be immediately treated for dengue, and the patients that test negative would still require subsequent diagnostic investigation. We recommend external validation in different settings.

## Abbreviations

CI: confidence interval
CNPq: Brazilian National Council of Scientific and Technological Development
D4: dengue 4
DENV-1: dengue virus serotype 1
DENV-2: dengue virus serotype 2
DENV-3: dengue virus serotype 3
DENV-4: dengue virus serotype 4
DNA: deoxyribonucleic acid
ELISA: enzyme-linked immunosorbent assay
FIOCRUZ: Oswaldo Cruz Foundation
IgG: immunoglobulin G
IgM: immunoglobulin M
INI-FIOCRUZ: Evandro Chagas National Institute of Infectious Diseases, Oswaldo Cruz Foundation
IQR: interquartile range
LR+: positive likelihood ratio
LR−: negative likelihood ratio
ND: non-dengue
NPV: negative predictive value
NS1: nonstructural protein-1
OR: odds ratio
PPV: positive predictive value
RNA: ribonucleic acid
RT-PCR: reverse transcriptase-polymerase chain reaction.
STARD: Standards for Reporting of Diagnostic Accuracy Study
UPA 24H: Unidade de Pronto Atendimento
WHO: World Health Organization
